# Causal Inference of the Effect of Vaccination on COVID-19 Disease Severity and Need for Intensive Care Unit Admission Among Hospitalized Patients in an African Setting

**DOI:** 10.7759/cureus.67719

**Published:** 2024-08-25

**Authors:** Eskedar Kebede Belayneh, Tigist Workneh Leulseged, Blen Solomon Teklu, Bersabel Hilawi Tewodros, Muluken Zeleke Megiso, Edengenet Solomon Weldesenbet, Mefthe Fikru Berhanu, Yohannes Shiferaw Shaweno, Kirubel Tesfaye Hailu

**Affiliations:** 1 Internal Medicine, Mulu-G Health Services, Addis Ababa, ETH; 2 Public Health, Medical Research Lounge (MRL), Addis Ababa, ETH; 3 Internal Medicine, St. Paul’s Hospital Millennium Medical College, Addis Ababa, ETH; 4 Internal Medicine, Atlas College of Health Sciences, Addis Ababa, ETH; 5 Internal Medicine, University Medical Care, Silver Spring, USA; 6 Internal Medicine, Sheba Medical Center, Tel Aviv, ISR; 7 Internal Medicine, Wachemo University, Hosanna, ETH; 8 Obstetrics and Gynaecology, Prolato Clinical Research Center, Houston, USA; 9 Otolaryngology, Adama General Hospital and Medical College, Adama, ETH; 10 Internal Medicine, Life Map Higher Learning Institute, Addis Ababa, ETH

**Keywords:** ethiopia, causal inference, retrospective cohort, vaccination, covid-19

## Abstract

Background

Coronavirus disease 2019 (COVID-19) is a novel, primarily respiratory, coronavirus that became a pandemic when it spread to over 210 countries and led to the death of over six million people. There is no definitive treatment for COVID-19, but vaccines have been developed that can help prevent severe illness and death. Studies have investigated the effect of vaccination on disease severity and outcome, and the findings indicate that vaccination is linked to a significant reduction in the risk of hospitalization, intensive care unit (ICU) admission, and disease mortality. However, there is a scarcity of evidence in Africa in general, and no similar study has been conducted in Ethiopia yet. Therefore, the study aimed to assess the effect of vaccination on COVID-19 disease severity and the need for ICU admission among hospitalized patients at a private specialty clinic in Ethiopia.

Methods

A retrospective cohort study was conducted among 126 patients with COVID-19, 41 vaccinated and 85 unvaccinated, who were hospitalized between September 2021 and May 2022. Data were summarized using frequency (percentage) and median (interquartile range (IQR)). To compare the characteristics of the two groups, Chi-square/Fisher's exact and Mann-Whitney U tests at p-values of ≤ 0.05 were used. To identify the effect of vaccination on COVID-19 disease severity, a marginal structural model (MSM) with an inverse probability weighting (IPW) approach using a robust Poisson regression model was fitted. Adjusted relative risk (ARR) and 95% confidence interval (CI) for ARR were used for interpreting the result.

Results

The cohort included groups that were comparable in terms of their sociodemographic and clinical characteristics. More than half of the participants were older than 60 years (n = 66, 52.4%), were males (n = 71, 56.3%), and had one or more comorbid illnesses (n = 66, 52.4%). At admission, 85 (67.5%) had severe disease, and 11 (8.7%) progressed after hospitalization and required ICU admission, of which three unvaccinated cases died. From the final model, vaccination was found to be associated with a 62% decreased risk of developing severe COVID-19 disease if infected, compared to not getting vaccinated (ARR = 0.38, 95% CI = 0.23-0.65, p < 0.0001).

Conclusions

The study’s findings support previous reports that vaccinated people are less likely to develop severe COVID-19 disease if later infected with the virus, emphasizing the importance of continuing efforts to promote COVID-19 vaccination not only to safeguard individuals but also to confer community-level immunity.

## Introduction

Coronavirus disease 2019 (COVID-19) is a respiratory illness caused by a virus called severe acute respiratory syndrome coronavirus 2 (SARS-CoV-2). The virus was first identified in Wuhan, China, in December 2019. Since then, it has spread to over 210 countries and territories, infected over 700 million people, and killed over six million. In Ethiopia, over 500,000 confirmed cases and over 7,000 deaths have been reported as of August 9, 2023 [1].

There is no specific treatment for COVID-19. Instead, supportive care is the mainstay of treatment, which includes conservative measures such as rest, fluids, and medications to relieve mild symptoms. In severe cases, patients may require oxygen therapy or mechanical ventilation. Additionally, novel agents such as antiviral medications, corticosteroids, and monoclonal antibodies have demonstrated efficacy in reducing disease severity and improving outcomes in specific patient populations [2, 3]. Therefore, as a more effective strategy to reduce the risk of mortal outcomes, the creation of a COVID-19 vaccination was expedited for primary prevention. The first COVID-19 vaccine, COMIRNATY® COVID-19 vaccine developed by Pfizer-BioNTech, was approved by the U.S. Food and Drug Administration on August 23, 2021 [4]. Since then, a number of vaccines have been developed by different companies, and millions of people around the world have been vaccinated. As of August 9, 2023, a total of 13,492,264,486 vaccine doses have been administered globally [1]. The Ethiopian Federal Ministry of Health launched COVID-19 immunization on March 13, 2021, and as of May 27, 2023, a total of 68,856,793 vaccine doses have been provided [1, 5]. AstraZeneca (Oxford University/AstraZeneca) was the first vaccine to be introduced to the country, but later, Sinopharm (Sinopharm), Johnson & Johnson/Janssen (Johnson & Johnson), and Pfizer-BioNTech (Pfizer-BioNTech) were used in addition to addressing a wider population base in the country, including the younger age groups.

The safety and efficacy of the vaccines have been proven in controlled studies and are expected to reduce morbidity and mortality. However, there is an irrefutable need for further evidence of the effectiveness of vaccines in preventing severe illness and death from COVID-19 in the real world [6-13]. With this in mind, a number of studies have investigated the effect of the COVID-19 vaccination on disease severity and outcome. According to these studies, vaccination has been linked to a significant reduction in the risk of hospitalization, intensive care unit (ICU) admission, and death from COVID-19 [14-20]. However, there is a scarcity of evidence throughout Africa in general, and no similar study has been conducted in Ethiopia yet [21,22]. Moreover, disease epidemiology in Africa appears to differ from that of the rest of the world thus far in terms of disease severity, hospitalization rate, and mortality rate [23-30]. Given these disparities in disease epidemiology and the lack of adequate evidence, it is especially important to understand the potential changes in disease patterns in the post-vaccination era in a local setting. Findings from such research can be used to make informed decisions at both the public health policy design and individual levels. Therefore, the aim of this study was to assess the effect of vaccination on COVID-19 disease severity and the need for ICU admission among hospitalized patients at a private specialty clinic in Ethiopia from September 2021 to May 2022.

This article was previously posted to the medRxiv preprint server on August 24, 2023.

## Materials and methods

Study setting and design

An institution-based retrospective cohort study, following the Strengthening the Reporting of Observational Studies in Epidemiology (STROBE) checklist for cohort studies, was conducted from June to August 2023 among COVID-19 patients who were admitted at a private clinic, Mulu-G Health Services, Addis Ababa, Ethiopia, between September 2021 and May 2022. The hospital is a primary internal medicine, gynecology, and pediatric specialty center, which had a dedicated ward for COVID-19 patient management during the pandemic. The cohort was classified into two groups based on their vaccination status for COVID-19: fully/partially vaccinated (exposed group) and unvaccinated (non-exposed group).

Population and sample size

The study included all laboratory-confirmed COVID-19 patients based on reverse transcription polymerase chain reaction (RT-PCR) tests who were admitted and completed their follow-up at the hospital between September 2021 and May 2022 and had complete data on basic clinical and laboratory parameters and COVID-19 vaccination status. Accordingly, from the 184 admissions, 126 eligible participants (41 vaccinated and 85 unvaccinated) were included in the study.

From the 41 vaccinated patients, data on the time between vaccination and hospitalization were documented for 35 cases. The average time between vaccination and hospitalization was 23 days (interquartile range (IQR): 21-60 days). Data on the type of vaccine were available for 30 of the vaccinated patients; all 30 received the AstraZeneca (Oxford University/AstraZeneca) vaccine. However, the type of vaccine was missing for the remaining 11 patients, as they were not sure of the name. Among the 30 AstraZeneca (Oxford University/AstraZeneca) vaccinated patients, 22 were hospitalized before receiving the second dose. Four patients had received both doses, while the number of doses was undocumented for the remaining four.

A post-hoc power analysis was calculated using G*Power statistical software (Ver. 3.19.4, Heinrich-Heine-Universität Düsseldorf, Düsseldorf, Germany) to check the power of the study using a two-tailed z-test for difference between two independent proportions with the following statistical parameters: 5% level of significance, proportion of severe COVID-19 and sample size in the vaccinated group of 36.6% and 41, respectively; and proportion of severe COVID-19 and sample size in the non-vaccinated group of 82.4% and 85, respectively. Finally, the power of the study was found to be 99.9%.

Operational definitions

Mild COVID-19 disease was characterized by fever, malaise, cough, upper respiratory symptoms, and/or less common features of COVID-19 (headache, loss of taste or smell, etc.) [2, 3]. Moderate COVID-19 disease was characterized by individuals who showed evidence of lower respiratory disease during clinical assessment or imaging and who could maintain oxygenation on room air [2, 3]. Severe COVID-19 disease included patients who had an oxygen saturation (SPO_2_) ≤ 93% on atmospheric air or a ratio of arterial partial pressure of oxygen to fraction of inspired oxygen (PaO_2_/FiO_2_) (SpO_2_/FiO_2_ (S/F) ratio < 315), in respiratory distress or respiratory rate > 30 breaths/minutes, or lung infiltrates involving > 50% of the lungs on a chest X-ray [2, 3].

Data collection procedures and quality assurance

Data on the sociodemographic, clinical, laboratory, and vaccination status of the patients was extracted from the electronic data registry system that was designed using the World Health Organization (WHO) Case Report Form (CRF) patient management and follow-up form. Data quality was assured through the proper training of two data collectors (general practitioners) on the tool, double data entry, and data cleaning by checking for inconsistencies, numerical errors, and missing parameters. All data management and analysis were performed using the 2021 STATA software version 17.0 (StataCorp LLC, College Station, TX).

Statistical analysis

The study population characteristics were summarized and presented using frequency with percentages for categorical variables. Symptom duration, measured in days, was summarized using a median with IQR due to the skewed distribution of the data (Kolmogorov-Smirnov and Shapiro-Wilk test p-value <0.0001). To compare the sociodemographic and clinical characteristics within the cohort (vaccinated vs. unvaccinated), a chi-square test was used. Whenever the assumption of a chi-square test where no cell should have expected a count of < 5 failed, Fisher's exact test was used instead. To compare the median symptom duration between the groups, a Mann-Whitney U test was used. For all tests, a statistically significant difference was detected for variables with a p-value of ≤ 0.05.

To identify the effect of vaccination on COVID-19 disease severity (moderate vs. severe), the marginal structural model (MSM) with inverse probability weighting (IPW) approach was used in two steps.

First, the treatment model using a binary logistic regression model was run to estimate the probability of vaccination status given the covariates (propensity score). The estimated probability of vaccination status was then used to compute the inverse probability weights for each individual. The inverse of the probability of vaccination was then used to weight each individual in the estimation of the marginal odds ratio. Univariate analysis at the 25% level of significance and clinical judgment was used to select variables to be included in the treatment model.

Subsequently, the final outcome model, using the vaccination status variable adjusted for inverse probability weights, was fitted to predict COVID-19 disease severity. To identify the effect of vaccination on disease severity, a robust Poisson regression model was selected. The fitness of the model was assessed using Pearson's chi-square and deviation tests, and the model fit the data well. Accordingly, on the final model, a p-value of ≤ 0.05 indicates that vaccination is a significant predictor of COVID-19 disease severity. In cases of significant relationships, adjusted relative risk (ARR) and 95% CI for ARR were used for interpreting the result. The effect of vaccination on the need for ICU admission was not assessed due to the small frequency of the outcome (only 11 cases required ICU admission).

## Results

Sociodemographic and clinical characteristics

More than half of the participants were older than 60 years (n = 66, 52.4%) and were males (n = 71, 56.3%). The frequent presenting symptom was cough in 119 (94.4%), followed by anorexia in 96 (76.2%), and constitutional symptoms of myalgia in 63 (50.0%), arthralgia in 62 (49.2%), and headache in 51 (40.5%). Anosmia and/or ageusia were reported by 58 (46.0%). The median duration of symptoms was seven days (IQR: four to 10 days), with a minimum of one day and a maximum of 20 days. Sixty-six patients (52.4%) presented with one or more comorbid conditions, with hypertension and diabetes being the most prevalent, affecting 38 patients (30.2%) each. 

According to the chi-square/Fisher's exact and Mann-Whitney U tests, there was no statistically significant difference in age, sex, presenting symptom, symptom duration, or comorbid illness history between vaccinated and unvaccinated patients (Table 1).

**Table 1:** Sociodemographic and clinical characteristics of the COVID-19 patients in the study group (n = 126) IQR: interquartile range; HIV: human immunodeficiency virus

Variable		Total (%)	COVID-19 vaccinated		p-value
			No (%)	Yes (%)	
Age (in years)	≤ 40	14 (11.1)	11 (12.9)	3 (7.3)	0.238
	40-60	46 (36.5)	34 (40.0)	12 (29.3)	
	≥ 60	66 (52.4)	40 (47.1)	26 (63.4)	
Sex	Female	55 (43.7)	33 (38.8)	22 (53.7)	0.116
	Male	71 (56.3)	52 (61.2)	19 (46.3)	
Cough	No	7 (5.6)	4 (4.7)	3 (7.3)	0.681
	Yes	119 (94.4)	81 (95.3)	38 (92.7)	
Shortness of breath	No	84 (66.7)	60 (70.6)	24 (58.5)	0.179
	Yes	42 (33.3)	25 (29.4)	17 (41.5)	
Chest pain	No	108 (85.7)	75 (88.2)	33 (80.5)	0.244
	Yes	18 (14.3)	10 (11.8)	8 (19.5)	
Fever	No	82 (65.1)	54 (63.5)	28 (68.3)	0.599
	Yes	44 (34.9)	31 (36.5)	13 (31.7)	
Headache	No	75 (59.5)	52 (61.2)	23 (56.1)	0.586
	Yes	51 (40.5)	33 (38.8)	18 (43.9)	
Arthralgia	No	64 (50.8)	47 (55.3)	17 (41.5)	0.146
	Yes	62 (49.2)	38 (44.7)	24 (58.5)	
Myalgia	No	63 (50.0)	45 (52.9)	18 (43.9)	0.342
	Yes	63 (50.0)	40 (47.1)	23 (56.1)	
Anorexia	No	30 (23.8)	21 (24.7)	9 (22.0)	0.734
	Yes	96 (76.2)	64 (75.3)	32 (78.0)	
Anosmia/ageusia	No	68 (54.0)	49 (57.6)	19 (46.3)	0.233
	Yes	58 (46.0)	36 (42.4)	22 (53.7)	
Diarrhea	No	115 (91.3)	76 (89.4)	39 (95.1)	0.501
	Yes	11 (8.7)	9 (10.6)	2 (4.9)	
vomiting	No	116 (92.1)	79 (92.9)	37 (90.2)	0.727
	Yes	10 (7.9)	6 (7.1)	4 (9.8)	
Nausea	No	113 (89.7)	79 (92.9)	34 (82.9)	0.117
	Yes	13 (10.3)	6 (7.1)	7 (17.1)	
Symptom duration (Median, IQR)		7.0 (4.0-10.0)	7.0 (4.5-10.0)	7.0 (4.0-9.0)	0.619
Diabetes	No	88 (69.8)	57 (67.1)	31 (75.6)	0.327
	Yes	38 (30.2)	28 (32.9)	10 (24.4)	
Hypertension	No	88 (69.8)	60 (70.6)	28 (68.3)	0.793
	Yes	38 (30.2)	25 (29.4)	13 (31.7)	
HIV	No	120 (95.2)	82 (96.5)	38 (92.7)	0.390
	Yes	6 (4.8)	3 (3.5)	3 (7.3)	
Asthma	No	123 (97.6)	82 (96.5)	41 (100.0)	0.550
	Yes	3 (2.4)	3 (3.5)	0	
Cardiac disease	No	120 (95.2)	82 (96.5)	38 (92.7)	0.390
	Yes	6 (4.8)	3 (3.5)	3 (7.3)	

Vital signs and laboratory parameters

Upon baseline assessment, only a smaller proportion of patients presented with abnormal vital signs and laboratory parameters. Of these, 27 (21.4%) had elevated systolic blood pressure (SBP), 18 (14.3%) had elevated diastolic blood pressure (DBP), 41 (32.5%) had an elevated pulse rate (PR), and nine (7.1%) had hyperthermia. Furthermore, 89 (70.6%) had an elevated neutrophil-to-lymphocyte ratio (NLR), 73 (57.9%) had an elevated hematocrit, 34 (27.0%) had a platelet count of less than 150 x 103/ul or greater than 450 x 103/ul, 29 (23.0%) had an elevated blood urea nitrogen (BUN) level, and 40 (31.7%) had an elevated creatinine level. On the other hand, the majority (n = 90, 71.4%) presented with hypothermia.

Additionally, the result showed that unvaccinated patients were more likely to have a raised pulse rate (33 (38.8%) vs. eight (19.5%), p-value=0.030), and BUN (24 (28.2%) vs. five (12.2%), p-value=0.045) than vaccinated patients. Otherwise, there were no significant differences between the two groups in terms of their blood pressure, oxygen saturation, temperature, NLR, hematocrit level, platelet count, or creatinine level (Table 2).

**Table 2:** Vital signs and laboratory parameters of the COVID-19 patients in the study group (n = 126) *statistically significant; SBP: systolic blood pressure; DBP: diastolic blood pressure; PR: pulse rate; NLR: neutrophil-to-lymphocyte ratio; BUN: blood urea nitrogen

Variable		Total (%)	COVID-19 vaccinated		p-value
			No (%)	Yes (%)	
SBP (mmHg)	< 140	99 (78.6)	70 (82.4)	29 (70.7)	0.136
	≥ 140	27 (21.4)	15 (17.6)	12 (29.3)	
DBP (mmHg)	< 90	108 (85.7)	74 (87.1)	34 (82.9)	0.535
	≥ 90	18 (14.3)	11 (12.9)	7 (17.1)	
PR (per minute)	<100	85 (67.5)	52 (61.2)	33 (80.5)	0.030*
	≥ 100	41 (32.5)	33 (38.8)	8 (19.5)	
Temperature (^o^C)	< 36.5	90 (71.4)	62 (72.9)	28 (68.3)	0.653
	36.5-37.5	27 (21.4)	18 (21.2)	9 (22.0)	
	> 37.5	9 (7.1)	5 (5.9)	4 (9.8)	
NLR	≤ 3	37 (29.4)	26 (30.6)	11 (26.8)	0.657
	3-6	35 (27.8)	22 (25.9)	13 (31.7)	
	6-9	26 (20.6)	16 (18.8)	10 (24.4)	
	>9	28 (22.2)	21 (24.7)	7 (17.1)	
Hematocrit (%)	≤ 45	53 (42.1)	35 (41.2)	18 (43.9)	0.772
	>45	73 (57.9)	50 (58.8)	23 (56.1)	
Platelet (x 10^3^/ul)	150-450	92 (73.0)	60 (70.6)	32 (78.0)	0.377
	<150/>450	34 (27.0)	25 (29.4)	9 (22.0)	
BUN (mg/dl)	< 20	97 (77.0)	61 (71.8)	36 (87.8)	0.045*
	≥ 20	29 (23.0)	24 (28.2)	5 (12.2)	
Creatinine (mg/dl)	<1.1	86 (68.3)	59 (69.4)	27 (65.9)	0.688
	≥ 1.1	40 (31.7)	26 (30.6)	14 (34.1)	

COVID-19 disease severity, the need for ICU admission, and outcomes

Of the 126 hospitalized COVID-19 patients, 41 (32.5%, 95% CI = 23.2-40.3%) had moderate disease, while 85 (67.5%, 95% CI = 59.7-76.8%) had severe disease upon admission. Among the 85 severe cases, 15 were vaccinated, and the remaining 70 were unvaccinated.

Disease progression after hospitalization that necessitated ICU care occurred in 11 cases (8.7%, 95% CI = 4.9-15.2%). Of these 11 cases, one was vaccinated and was discharged and recovered. The remaining 10 were unvaccinated, of which seven recovered and three died. Of the remaining 123 cases, 82 out of 85 unvaccinated cases and all 41 vaccinated cases were discharged and improved.

Treatment model: logistic regression of factors associated with COVID-19 vaccination

The treatment model using a binary logistic regression model was run by including the following categories: age category, sex, fever, headache, arthralgia, anorexia, anosmia/ageusia, diarrhea, vomiting, nausea, diabetes, hypertension, SBP, DBP, PR, temperature, NLR, hematocrit, platelet, BUN, and creatinine. By fitting the final treatment model, the propensity score was estimated, and it was used to compute the inverse probability weights for each individual. The inverse of the probability of vaccination status was then used to weigh each individual in the estimation of the marginal odds ratio (Table 3).

**Table 3:** Binary logistic regression model of factors associated with vaccination status among COVID-19 patients in the study group (n = 126) AOR: adjusted odds ratio; CI: confidence interval; SBP: systolic blood pressure; DBP: diastolic blood pressure; PR: pulse rate; NLR: neutrophil-to-lymphocyte ratio; BUN: blood urea nitrogen; *statistically significant

Variable	AOR	95% CI AOR	p-value
Age, in years (R: ≤ 40)			
40-60	1.45	0.20, 10.51	0.715
≥ 60	3.16	0.40, 24.85	0.275
Sex (Male)	0.58	0.20, 1.69	0.316
Fever	0.94	0.26, 3.36	0.924
Headache	1.68	0.56, 5.06	0.356
Arthralgia	2.57	0.64, 10.32	0.182
Myalgia	0.65	0.16, 2.66	0.552
Anorexia	0.29	0.07, 1.15	0.078
Anosmia/ageusia	1.73	0.52, 5.74	0.367
Diarrhea	0.12	0.01, 1.28	0.080
Vomiting	1.00	0.11, 9.27	0.998
Nausea	5.49	0.88, 34.14	0.068
Diabetes	0.76	0.25, 2.28	0.624
Hypertension	0.93	0.28, 3.11	0.902
SBP (≥ 140 mmHg)	3.55	0.89, 14.15	0.072
DBP (≥ 90 mmHg)	0.89	0.17, 4.69	0.888
PR (≥ 100 /minute)	0.24	0.07, 0.77	0.017
Temperature (R: <36.5^o^C)			
36.5-37.5 oC	2.61	0.73, 9.31	0.140
>37.5 oC	2.84	0.31, 25.94	0.355
NLR (R: ≤ 3.00)			
3.01-5.99	1.22	0.32, 4.69	0.772
6.00-9.00	1.23	0.29, 5.19	0.774
≥ 9.01	0.58	0.11, 3.18	0.532
Hematocrit (> 45%)	1.40	0.47, 4.18	0.543
Platelet (<150/>450 x 103/ul)	0.64	0.21, 1.97	0.438
BUN (≥ 20 mg/dl)	0.20	0.04, 0.90	0.036
Creatinine (≥ 1.1 mg/dl)	2.22	0.63, 7.84	0.215

Outcome model: effect of vaccination on COVID-19 disease severity

To identify the effect of vaccination on COVID-19 disease severity, a robust Poisson regression model was run by fitting the vaccination status variable adjusted for inverse probability weights that were obtained from the treatment model above.

The result shows that vaccination is associated with a 62% decreased risk of developing severe COVID-19 disease if infected, compared to not getting vaccinated (ARR = 0.38, 95% CI = 0.23-0.65, p<0.0001) (Table 4).

**Table 4:** Robust Poisson regression of the effect of vaccination on disease severity among COVID-19 patients in the study group (n = 126) ARR: adjusted relative risk; CI: confidence interval; *statistically significant

Vaccination	COVID-19 severity		ARR	95% CI for ARR	p-value
	Moderate	Severe			
Yes	26	15	0.38	0.23, 0.65	<0.0001*
No	15	70	1	1	

## Discussion

This retrospective cohort study investigated the effect of vaccination on COVID-19 disease severity among hospitalized patients who were followed at a private specialty clinic in Ethiopia between September 2021 and May 2022. Although multiple studies are done internationally, this is the first of its kind to answer this research question at an Ethiopian institution. The study included 126 hospitalized COVID-19 patients, 41 vaccinated and 85 unvaccinated. The participants were fairly comparable in terms of their sociodemographic and clinical characteristics. More than half of the participants were older than 60 years (n = 66, 52.4%), were males (n = 71, 56.3%), and had one or more comorbid illnesses (n = 66, 52.4%). At admission, 85 (67.5%) had severe disease, and 11 (8.7%) progressed after hospitalization and required ICU admission. Three unvaccinated cases lead to mortality.

According to the robust Poisson regression analysis using the MSM model with the IPW approach, vaccination is causally associated with disease severity and progression in a protective way, with vaccinated people 62% less likely to develop severe COVID-19 disease if infected with the virus. The findings indicate that immunization plays a significant role in preventing the development of severe disease. This is a noteworthy finding, as it suggests that vaccination can help to protect people from the most serious consequences of COVID-19: hospitalization, long-term complications, and even death. Furthermore, it also contributes to the greater goal of reducing overall healthcare costs and preventing the spread of illness. The study's findings are consistent with the results of other studies that have shown vaccination to be effective in preventing severe COVID-19 outcomes in both Africa [21,22] and abroad [14-20]. Although the study findings note the importance of vaccination in reducing morbidity and mortality, a significant portion of the population in Ethiopia remains unvaccinated [1,5]. Hence, the importance of vaccination in reducing morbidity and mortality in combination with other preventative measures is a crucial instrument in advertising and supporting against COVID-19 and should not be overlooked. 

There are several strengths and limitations of the current study. The study's strengths include the fact that it is the first in Ethiopia and one of only a handful in Africa. It was conducted in a hospital setting where a more complete picture of the influence of the vaccine on those who were already infected and required admission could be studied. Additionally, the causal effect of vaccination was estimated using a causal inference model, which can provide strong evidence of causality from observational data. Its limitations are that further investigation of the vaccine effect in terms of type, dose, and period between vaccination and initial symptom could not be made since they were not fully documented. The generalizability is limited as it is a single-center study.

## Conclusions

The study shows that vaccinated people are less likely to develop severe COVID-19 disease if infected with the virus. The findings are consistent with studies conducted during clinical trials for COVID-19 vaccine development and post-market surveillance. These findings emphasize the importance of continued efforts to promote COVID-19 vaccination not only to safeguard individuals but also to confer community-level immunity. We recommend large prospective community and multicenter studies to gain a better understanding of the effect of vaccination on short- and long-term disease outcomes, taking into account the dose and type of vaccine.
